# The “Janus Face” of Platelets in Cancer

**DOI:** 10.3390/ijms21030788

**Published:** 2020-01-25

**Authors:** Maria Valeria Catani, Isabella Savini, Valentina Tullio, Valeria Gasperi

**Affiliations:** Department of Experimental Medicine, Tor Vergata University of Rome, 00133 Rome, Italy; savini@uniroma2.it (I.S.); valentinatullio.nu@gmail.com (V.T.)

**Keywords:** microvesicles, miRNAs, paraneoplastic thrombocytosis and thrombocytopenia, platelet activation, platelet-derived bioactive molecules, platelet-tumor crosstalk

## Abstract

Besides their vital role in hemostasis and thrombosis, platelets are also recognized to be involved in cancer, where they play an unexpected central role: They actively influence cancer cell behavior, but, on the other hand, platelet physiology and phenotype are impacted by tumor cells. The existence of this platelet-cancer loop is supported by a large number of experimental and human studies reporting an association between alterations in platelet number and functions and cancer, often in a way dependent on patient, cancer type and treatment. Herein, we shall report on an update on platelet-cancer relationships, with a particular emphasis on how platelets might exert either a protective or a deleterious action in all steps of cancer progression. To this end, we will describe the impact of (i) platelet count, (ii) bioactive molecules secreted upon platelet activation, and (iii) microvesicle-derived miRNAs on cancer behavior. Potential explanations of conflicting results are also reported: Both intrinsic (heterogeneity in platelet-derived bioactive molecules with either inhibitory or stimulatory properties; features of cancer cell types, such as aggressiveness and/or tumour stage) and extrinsic (heterogeneous characteristics of cancer patients, study design and sample preparation) factors, together with other confounding elements, contribute to “the Janus face” of platelets in cancer. Given the difficulty to establish the univocal role of platelets in a tumor, a better understanding of their exact contribution is warranted, in order to identify an efficient therapeutic strategy for cancer management, as well as for better prevention, screening and risk assessment protocols.

## 1. Introduction

Platelets were described for the first time in 1882, when the Italian pathologist Giulio Bizzozero identified in the blood vessels “very thin platelets, disc-shaped, with parallel surfaces or rarely lens-shaped structures, round or oval and with a diameter 2–3 times smaller than the diameter of the red cells…” that “when they are circulating in the blood stream of a living animal a small injury to the vessel wall, or contact with a foreign body is sufficient for them to become viscous, to adhere to one another and so form a white thrombus” [1]. Platelets, indeed, are small, anucleated cytoplasmic fragments derived from large (30–100 μm) progenitor cells, the megakaryocytes, formed in bone marrow, lungs and blood [2,3,4,5], which are vitally involved in thrombosis and hemostasis [6]. Nevertheless, besides this primary function, platelets are also recognized to affect immune and inflammatory responses, thus participating in regulation of biological mechanisms underlying a broad range of human disorders. A large body of experimental and clinical evidences, indeed, shows that platelet activation and dysfunction are implicated in diabetes, cardiovascular disease, chronic back pain, sepsis, Alzheimer’s disease, multiple sclerosis, psychiatric disorders and other central and peripheral pathological conditions [7,8,9,10,11]. In this context, the unexpected central role of platelets in cancer biology is noteworthy: They actively influence cancer cell behavior, but, on the other hand, platelet physiology and phenotype are impacted by tumor cells [12]. Indeed, a large number of experimental and human investigations support the hypothesis that tumor cells are able to modulate the RNA profile, number and activity of platelets that, once “educated”, would regulate the tumor microenvironment and progression in a way dependent on the patient, cancer type and treatment. Nonetheless, the exact molecular mechanisms underlying this platelet-cancer loop are not yet well defined, often due to contradictory data.

Therefore, based on this background, the present review will focus on platelet-cancer crosstalk and their mutual impact, especially considering how platelets might exert either a protective or a deleterious role in all steps of cancer progression.

## 2. Paraneoplastic Thrombocytosis and Thrombocytopenia in Cancer

In healthy subjects, platelet count ranges from 150,000 to 450,000/µL, with age-, sex-, race- and genetic background-specific reference intervals [13,14]. A fine-tuned control of both platelet number and function exists, being ensured by the delicate balance among their (i) production, (ii) maintenance in the circulation (average life span of 8–10 days) and (iii) clearance of senescent cells (via hepatic and splenic macrophages, as well by apoptosis) [15,16,17].

In the light of the central roles played by platelets in a multitude of biological events, abnormalities in their number that often accompany various pathologies are clearly relevant, and this also applies to cancer.

Although, with some inter-individual variations, a platelet count of ≥450 × 10^9^/L is a generally accepted value used to identify a clinically significant thrombocytosis [18], which has a multitude of potential etiologies. In particular, it can be classified as (i) primary thrombocytosis, when it occurs as the result of genetic or chronic myeloid disorders [19,20,21] or (ii) secondary or reactive thrombocytosis, when it occurs as a comorbidity of another underlying disease independent of a vascular event, including cancer. In the latter case, the pathology is called paraneoplastic thrombocytosis [22,23]. According to the clinical evidence that patients with a high platelet count have a higher risk to develop venous thromboembolism (VTE) [24], cancer patients frequently show activated coagulation pathways, resulting in a four-fold increase in thrombosis risk [25].

The first evidence of paraneoplastic thrombocytosis dates back to 1964, when Levin and Conley found that, among their hospitalized cancer patients, at least 40% had thrombocytosis [26]. Since then, an ever-growing body of studies has reported a significant association between thrombocytosis and solid tumors, with a range of thrombocytosis incidence at initial diagnosis of 4–55% [27,28,29,30,31]. This evidence may assume clinical implications, if we consider that a large amount of retrospective and meta-analysis studies point out to the correlation among higher platelet count and tumor progression, advanced-stage disease, vascular thromboembolic complications and poor survival in patients with different solid tumors, such as esophageal cancer, bladder cancer, inflammatory breast cancer and epithelial ovarian cancers (see Table 1) [28,31,32,33,34,35,36,37,38].

Conversely, such a correlation has not been found by other authors [29,39,40,41], or it appears strictly dependent on inflammatory components, as described by a recent retrospective study of 3654 patients with stage I–III breast cancer, of whom 6.5% had a diagnosis of Inflammatory Breast Cancer (IBC), the most aggressive form of breast tumors [37]. What emerged from this study is that thrombocytosis, more prevalent in IBC patients, correlated with poor overall survival in these subjects, but not in non-IBC individuals [37].

It must not be overlooked that some investigations reporting a correlation among platelet count, metastasis and shortened survival had some limitations, such as a low platelet count threshold (<200 × 10^9^/L) not clinically correct to define a patient as having real thrombocytosis [33,34,37,42]. In addition, heterogeneity in sample size, clinical stages, treatment and follow-up, smoking history and inclusion/exclusion criteria may make it difficult to establish a univocal association between thrombocytosis and poor prognosis in cancer patients.

**Table 1:** Main findings on the relationship between high platelet count ^1^ and cancer.

Cancer	Study	Platelet Cut-Off	Main Findings	Ref.
**Oesophageal**	- Retrospective - 584 adenocarcinoma patients with or without pre-operative neoadjuvant chemo-radiation therapy - 2.4% with high PLT	450 × 10^9^/L	- Death rates: 50% with normal PLT (median survival time: 76.9 months), 86% with high PLT (median survival time: 23.2 months) - No differences in age, gender, tumor T or N stages - Median survival time in patients without neoadjuvant therapy: 35.8 months with high PLT, 112 months with normal PLT (HR = 3.02, p = 0.032) - Median survival time in patients with neoadjuvant therapy: 16.2 months with high PLT, 52.1 months with normal PLT (HR = 2.31, p = 0.021)	[43]
	- Retrospective - 374 squamous cell carcinoma patients with non-neoadjuvant therapy - 21.1% with high PLT	293 × 10^9^/L	- PLT increased in patients with large and deep tumors, nodal involvement, and distant metastasis - CRP levels increased in patients with high PLT (p = 0.001) - Worse survival in patients with high PLT, especially in advanced tumor stage patients	[33]
	- Retrospective - 425 squamous cell carcinoma patients subjected to esophagectomy - 48.4% with high PLT	205 × 10^9^/L	- Overall 5-year survival: 60.7% with PLT below cut off value, 31.6% with PLT above cut off value (p < 0.001) - 5-year survival with no involvement of nodes: similar rates independent of PLT - 5-year survival with involvement of nodes: 32.0% with PLT below cut off value, 12.7% with PLT above cut off value (p = 0.004)	[34]
	- Retrospective - 119 squamous cell carcinoma patients subjected to esophagectomy - 20.2% with high PLT	300 × 10^9^/L	- No association between high PLT and disease-free (HR = 0.918, 95% CI = 0.524 − 1.608, p = 0.765) or overall (HR = 1.072, 95% CI = 0.618 − 1.891, p = 0.809) survival	[41]
	- Retrospective - 112 patients subjected to esophagectomy - 4% with high PLT	400 × 10^9^/L	- No correlation between PLT and patient survival (p < 0.644)	[39]
	- Retrospective - 381 patients (93% squamous cell carcinoma and 7% adenocarcinoma) subjected to esophagectomy - 3.4% with high PLT	400 × 10^9^/L	- Higher PLT in patients with adenocarcinoma (p = 0.003) - No correlation between PLT and prognostic factors - No correlation among PLT, site and degree of tumor penetration, lymph node involvement, distant metastasis, degree of differentiation, vascular, lymphatic and perineural invasion, presence of multiple cancers	[40]
**Cervical**	- Meta-analysis - 6521 patients	400 × 10^9^/L (seven studies) 300 × 10^9^/L (six studies) 200 × 10^9^/L (five studies) Not specified (one study)	- High PLT before surgery and/or chemotherapy associated with poor overall (HR = 1.50, 95% CI = 1.19 − 1.88, p = 0.001), progression-free (HR = 1.33, 95% CI = 1.07 − 1.64, p = 0.010) and recurrence-free (HR = 1.66, 95% CI = 1.20 − 2.28, p = 0.002) survival	[32]
**Epithelial ovarian**	- Retrospective - 619 patients - 31% with high PLT	450 × 10^9^/L	- High PLT associated with advanced-stage disease, vascular thromboembolic complications, higher preoperative levels of cancer antigen 125 and shortened survival	[28]
**Lung**	- Retrospective - 234 patients with Stage I non-small cell lung cancer	300 × 10^9^/L	- Correlation among PLT, disease progression (HR = 5.314, 95% CI = 2.750 − 10.269, p < 0.05) and death (HR = 3.139, 95% CI = 1.227 − 8.034, p < 0.05)	[31]
	- Meta-analysis - 5884 patients - 6.9–58.5% with high PLT	400 × 10^9^/L (6 studies) 300 × 10^9^/L (5 studies) 214.5 × 10^9^/L (1 study)	- High PLT associated with overall survival (HR = 1.74, 95% CI = 1.39-2.19, p < 0.001), advanced TNM stage (OR = 2.65, 95% CI = 1.77 − 3.97, p = 0.367), smoking history (OR = 2.70, 95% CI = 1.79 − 4.08, p = 0.373) - No correlation between high PLT associated and squamous cell carcinoma (OR = 1.54, 95% CI = 0.77 − 3.07, p = 0.017)	[29]

CI: confidence interval; CRP: C-reactive protein; HR: hazard ratio; OR: odds ratio; PLT: platelet count. ^1^ With respect to the cut-off values set by the authors.

However, available literature data suggest that thrombocytosis is a paraneoplastic event not depending on elongation of platelet half-life survival [44], but on increased thrombopoietin (TPO)-dependent thrombopoiesis, together with the action of inflammatory cancer-derived cytokines. TPO is normally produced and secreted by the liver, kidney and bone marrow at a fixed rate and it promotes megakaryocyte growth and platelet generation, by binding to its receptor c-MPL and triggering activation of the Janus kinase (JAK)/signal transducer and activator of the transcription (STAT) pathway [45]. It is well documented that increased circulating TPO levels might be one of the mechanisms accounting for cancer-related thrombocytosis, as demonstrated by elevated TPO levels in plasma of cancer patients with a high platelet count [46,47,48]. Two different and complementary mechanisms have been proposed, both encompassing tumors that represent a TPO source per se and, moreover, secrete factors targeting hepatic TPO synthesis. In particular, it has been reported that certain cancer cells, besides expressing TPO receptors on their surface [49], are also able to produce and release functional TPO [47,50], thus contributing to the rise in blood TPO levels. Additionally, cancer cells release a plethora of humoral factors and cytokines, and some of them have been shown to upregulate hepatic TPO biosynthesis; this is the case of the pleiotropic cytokine interleukin (IL)-6, a major mediator of inflammation and activator of STAT3 [51], whose deregulated overexpression has been associated with tumor progression [28,52,53,54,55]. Both IL-6 and its receptors (IL-6R and sIL-6R) are, indeed, upregulated in tumors [56,57,58,59,60,61] and their increased content in plasma of cancer patients correlates with a poor diagnosis [28,56,61,62], thus indicating clinical utility of IL-6 as a biomarker or therapeutic target in cancer management. An elegant model proposed by several authors suggests that IL-6 plays a crucial role in inducing cancer-related thrombocytosis, via up-regulation of hepatic TPO transcription [28,53,54,55]. In particular, this molecular model hypothesizes that cancer cells release large amounts of IL-6 that, in turn, determines complex chains of events (i.e., an increase in platelet count, tumor growth and metastasis) reinforcing themselves through a feed-forward loop. This hypothesis has been confirmed by the study of Stone and co-workers [28], who analyzed 619 patients with epithelial ovarian cancer and of whom 30% had thrombocytosis at the time of initial diagnosis. The researchers found that TPO and IL-6 levels were high in patients who had thrombocytosis, as compared with those who did not, and that an increase in IL-6 levels positively correlated with plasma TPO levels and thrombocytosis, while negatively correlating with patient survival. Further proofs of the crucial role of IL-6 in paraneoplastic thrombocytosis have also been provided by molecular/genetic and pharmacological experiments: Silencing of *Il-6* and *tpo* genes fully abrogated thrombocytosis in murine ovarian cancer, and siltuximab (humanized anti-IL-6 antibody) significantly reduced tumor growth and platelet count, both in murine and human ovarian cancers [28].

Other circulating factors released by cancer cells and known to stimulate thrombopoiesis and megakaryopoiesis are granulocyte colony-stimulating factor (G-CSF) and granulocyte-macrophage colony-stimulating factor (GM-CSF), whose blood levels are increased in cancer patients with thrombocytosis [63]. 

A more in-depth analysis of basal cytokine profile in 81 newly diagnosed IBC patients revealed that patients with thrombocytosis, although not differing in IL-6 levels with respect to IBC subjects without thrombocytosis, showed a positive correlation between serum levels of Growth-Regulated Oncogene (GRO) and Transforming Growth Factor (TGF)-β and IBC-related thrombocytosis [37]. In this context, it should be underlined that both cancer cells and activated platelets are able to release GRO and TGF-β [64,65,66], thus suggesting that the observed increase in their content might be a consequence rather than a cause of thrombocytosis. In addition, the study has several limitations, above all the lowering of the thrombocytosis threshold from 450 to 300 × 10^9^/L. Therefore, more studies are needed to establish a real relationship between these two cytokines and platelets in the context of tumor biology. 

While thrombocytosis is more frequently reported to be associated with increased mortality, some findings also suggest the presence of cancer-related thrombocytopenia. For example, a strong trend toward increased mortality has been found in thrombocytopenic patients (hazard ratio (HR) = 1.50, but without reaching statistical significance) [43], although it is conceivable that thrombocytopenia might be a surrogate for general debility and/or other clinical factors, such as possible sepsis and hematological abnormalities that could contribute to overall mortality.

Thrombocytopenia is a frequent complication in solid tumors [67]. The degree and incidence of this disease depends on the type of malignancy, tumor stage and treatment approach [68]. It has also been described as a complement of local cancer recurrence and may be considered a paraneoplastic syndrome [69] Some tumors can alter the platelet count below 100 × 10^9^/L, leading to thrombocytopenia, and therefore cancer patients have a high risk of hemorrhagic complications [68]. The first evidence of low platelet count and bleeding episodes in patients with malignancies came from Gaydos in 1962: He demonstrated that bleeding episodes in patients with leukemia were frequently associated with a decreased platelet count [70]. Since then, other studies reported similar bleeding events in solid tumor patients [71,72]. 

Single nucleotide polymorphisms (SNPs) and mutations in genes encoding for cytokines and transcription factors are both two major causes of thrombocytopenia in solid tumors, including lung, breast, ovary and colorectal cancers [72]. Just as an example, the -31 T > C SNP of the *il-1β* gene was up-regulated in solid tumors associated with thrombocytopenia [73,74]. It is unclear how IL-1β can induce thrombocytopenia in solid tumors, but it is known that -31 T > C SNP can increase susceptibility to thrombocytopenia in these malignancies [72]. A strong association between IL-1β -31 T > C SNP and *Helicobacter pylori* infection has also been reported: The two phenomena collaborate themselves to increase the risk of gastric cancer with hemorrhagic complications [75,76]. IL-6 is also involved in thrombocytopenic mechanisms, as well as in paraneoplastic thrombocytosis. The IL-6 -174 G/C SNP has been reported in many malignancies, including adenocarcinoma, lung, colorectal, gastric and ovarian cancers [73,77,78]. This polymorphism also has been correlated with a poor prognosis, because it can induce antibody production against platelets, increasing the risk of thrombocytopenia [72].

As mentioned before, several transcription factors can be associated with thrombocytopenia, due to their involvement in platelet production. For example, overexpression of GATA3, a member of the GATA family of transcription factors that control maturation of hematopoietic stem cells, can stimulate platelet clearance, worsening the prognosis in breast cancer [79]. Another example is represented by Homeobox (HOA) genes that control proliferation and maturation of hematopoietic stem cells: Hypermethylation of *hoxa11*, for example, increased the incidence of thrombocytopenia and risk of poor prognosis in lung, gastric and breast cancers [80,81]. Although few studies have evaluated genetic changes in the incidence of thrombocytopenia, it seems that investigations on the mechanisms accounting for this phenomenon may be useful for the prevention of bleeding in solid tumors and for choosing the appropriate treatment [72].

Clinical observations of the impact of platelet count on cancer biology have been supported by studies employing platelet-depleted or transgenic mice [82]. A significant reduction in neovascularization has been observed in different transgenic mice-rendered thrombocytopenia [83], with GPIbα/IL4R transgenic mice (lacking the receptor for the von Willebrand factor as well as other adhesive and pro-coagulant proteins) showing the most severe phenotype [83,84]. Furthermore, in a mammary carcinoma murine model, platelet depletion increased the efficacy of the chemotherapy, by favoring drug delivery and tumoricidal action [85]. Thus, currently available animal models of platelet dysfunction may provide a framework for better understanding the molecular mechanisms through which thrombocytosis or thrombocytopenia impact cancer progression.

## 3. Platelet-Derived Bioactive Compounds

It is now recognized that tumor cells and platelets strictly influence each other, thus establishing the so-called “platelet-tumor loop”. The need for recruiting platelets is supported by several evidences: (i) tumor cells sequester platelets in order to escape themselves from immune system surveillance [86,87]; (ii) platelets associate with cancer emboli, thus prolonging survival of circulating tumor cells and promoting their arrest and adhesion to endothelium for transmigrating to metastatic sites [86,88]; and (iii) platelets secrete a plethora of tumor, angiogenic, growth and permeability factors, which can regulate tumor growth, epithelial to mesenchymal transition and metastasis (see below). Noteworthy, circulating hyperactivated platelets, as well as exhausted platelets (i.e., with totally or partially depleted granules, as a consequence of previous activation), are commonly found in subjects with different tumor types, concomitant with the high incidence of VTE [89].

In order to grow and develop metastasis, tumor cells concurrently influence platelet behavior by up-regulating synthesis and/or release of several compounds able to promote platelet activation and aggregation [90,91,92]. For example, when compared to benign tumors, malignant cells show higher generation of thrombin, one of the most potent platelet activators with strong pro-coagulating properties [93], but also able (when bound to thrombomodulin expressed on endothelium) to attenuate the thrombotic cascade [94,95]. Accordingly, blood thrombin concentrations negatively predict both success to surgery/chemotherapy and survival of patients with gynecological tumors [96]. A cohort study enrolling 112 patients with different cancers revealed that, although without association with disease state, rise in thrombin levels were dependent on tumor site, with lung cancers having more significant increases compared to brain and pancreas cancers [97]. Detrimental effects of increased thrombin generation seem to rely on its ability in promoting, in concert with its targets (among them, protease-activated receptor-1 and -4), cancer adhesion to platelets or endothelium via up-regulation of pro-inflammatory cytokines, adhesion molecules, angiogenic factors, and matrix-degrading proteases, thereby dramatically increasing tumor growth, angiogenesis, invasion, and metastasis [98]. Proofs of the “tumor cell-induced platelet aggregation” also come from quantitative analysis of circulating neutrophil elastase (which proteolytically activates integrin αIIbβ3) and serglycin (a pro-apoptotic and half-life cytokine regulating protein) in melanoma patients; while levels of the former were found up-regulated in cancer subjects, content of the latter was low [99], thus indicating the role of activated platelets in promoting cancer progression.

Although data on the molecular mechanisms underlying platelet hyperactivation are still not well-defined and often contradictory, nonetheless they suggest the involvement of some platelet bioactive molecules. Among them, those contained in α-granules play a pivotal role during platelet-cancer crosstalk (Table 2).

Besides P-selectin and other clotting proteins (such as thrombospondin, fibrinogen, fibronectin integrin αIIβ3, integrin αVβ3, factor V, and the von Willebrand factor), α-granules contain growth and pro-angiogenic factors (including Platelet-Derived Growth Factor (PDGF), Vascular Endothelial Growth Factor (VEGF), TGF-β, Epidermal Growth Factor (EGF) and Angiopoetin-1 (Ang-1)), which are secreted following platelet activation and, either directly or indirectly, promote tumorigenesis. For example, besides directly promoting cancer progression and tumor cell extravasation (by facilitating interaction of cancer cells with platelets and endothelium) [109,110], P-selectin may indirectly exacerbate cancer evolution by triggering thrombin generation [111] and rapid monocyte exposure of tissue factor (TF) [100]. The latter protein, primarily involved in activation of the clotting cascade, positively affects tumor growth and metastasis: TF (produced by cancer cells) present in the tumor microenvironment may increase cell survival and/or angiogenesis, while TF present in the bloodstream (deriving from both monocytes and circulating cancer cells) has been shown to enhance thrombosis, tumor growth and metastasis. Several studies showed that P-selectin (either soluble or membrane-bound) changes in cancer patients, although controversial data have been documented. Some clinical studies found high P-selectin levels in cancer patients, with or without correlation to tumor clinical advancement [97,112]; conversely, recent observational and longitudinal studies enrolling patients with heterogeneous cancers found a decrease in platelet surface expression of this protein, which, together with diminished integrin αIIbβ3 exposure, thrombin and collagen receptor responsiveness and monocyte–platelet aggregate formation, correlates with risk of mortality and VTE [113,114]. Nevertheless, a lack of data on platelet activation changes in relation to cancer evolution makes it difficult to clarify whether decreased platelet reactivity is a consequence of continuous pre-activation in patients with poor prognosis or, rather, it represents a cancer-independent event, or even it is the result of a lack of protective effects exerted by activated platelets.

PDGF also has been implicated in tumorigenesis: It acts on tumor cells, thereby favoring proliferation, survival and invasion [104,105], and, in the meanwhile, creates a favorable microenvironment for tumor cells by inducing changes into tumor stroma and promoting blood vessel maturation (especially in advanced stages of angiogenesis) [115]. Accordingly, anti-PDGF drugs significantly inhibit tumor growth and metastasis, although downregulation of PDGF-BB (one of the five isoforms) signaling is associated with tumor cell dissemination and metastasis [106]. In the latter case, a protective role of PDGF-BB has been suggested, since its overexpression, by increasing tumor pericyte content, decreases colorectal and pancreatic cancer growth [116]. However, data on PDGF-BB content in cancer patients are controversial: Concentrations of secreted PDGF-BB, for example, have been found to be significantly high in the serum of colorectal carcinoma patients [117], but low in liver cancer patients with recurrence [118].

Among platelet-derived growth factors/cytokines, VEGF, EGF and Ang-1 play a crucial role in cancer angiogenesis [101,102,103]. Concerning the pro-angiogenic effect, the mutual influence of platelets and tumor cells also is not fully determined. A recent study performed on twenty-four women with active breast cancer and ten healthy controls showed that breast cancer and its chemotherapeutic treatment influence platelet phenotype, by increasing VEGF release and modulating the response to antiplatelet therapy [119]. Noticeably, an autocrine–paracrine loop, occurring in the bone marrow microenvironment and involving VEGFR-1-dependent megakaryocyte maturation has been documented [120]. Such evidence, together with the finding that tumor-derived IL-6 leads to enhanced megakaryocyte VEGF expression and a higher platelet VEGF load (concomitantly associated with fast tumor growth kinetics and poor diagnosis), strongly suggest a cooperation between platelets and cancer in promoting angiogenesis [121]. Conversely, low EGF levels have been found in cancer subjects with recurrence, and an inverse correlation between its concentrations and survival [118] has been documented in these subjects. Low serum Ang-1 levels, related to poor diagnosis, were also found in subjects with certain types of cancer [118]. On the contrary, patients with lung and ovarian cancer show high Ang-1 concentrations not related to patient survival [122].

In proteomic studies, α-granules also have been shown to contain a plethora of angiogenesis inhibitors, including endostatin, platelet factor-4, thrombospondin-1, α_2_-macroglobulin, plasminogen activator inhibitor-1 and angiostatin. Therefore, activated platelets can organize regulatory proteins in the α-granules in order to selectively address the release of pro- or anti-angiogenic factors, depending on which different sets of α-granules are segregated [107,108]. Although cancer cells might have the ability to provoke preferential release of pro-angiogenic mediators from platelet α-granules, in order to create a dynamic microenvironment favorable for their growth and survival [107], several experimental and clinical data point out that platelet secretion in the tumorigenic microenvironment might also be oriented towards an anti-angiogenic effect. Such a hypothesis is supported by several findings: (i) significantly higher endostatin levels have been found in hepatocellular carcinoma patients, as well as in gastric cancer subjects [123,124]; (ii) increases in circulating thrombospondin-1 have been found to positively correlate with survival of patients with gynecological and non-small cell lung cancer [125,126]; and (iii) higher levels of angiostatin have been found in serum of prostate cancer patients [127], as well as in urine of patients with epithelial ovarian cancer [128].

Platelets also secrete other factors, which can interfere with all steps of cancer development and metastasis. Serotonin, a monoamine synthesized by enterochromaffin cells in the intestinal mucosa, is largely (about 95%) sequestered by platelets in dense granules, from which it is released in response to various stimuli [12]. A large body of experimental data support both stimulatory and inhibitory properties of serotonin on tumor onset and progression [129,130,131,132,133,134]. Such different behavior is much likely due to the ability of serotonin to act in a concentration-dependent manner, as well as in its capability to activate distinct signaling pathways, depending on the receptor subtype present at various tumor stages. Coherently, high serotonin levels, correlating with the tumor stage, distant metastases and a poor prognosis, have been found in the serum of patients with different solid tumors [131,135,136,137], whereas low concentrations have been found in hepatocellular carcinoma patients, who showed recurrence after partial hepatectomy [118]; in untreated breast adenocarcinoma or malignant melanoma patients, where the phenotype (also associated with a high ATP/ADP ratio and index of delta storage pool deficiency) was more marked in regionally spread malignant tumors [138].

Platelets are also important producers of eicosanoids, lipids derived from polyunsaturated fatty acids (PUFAs), through the catalytic action of cyclooxygenase (COX) and lipoxygenase (LOX). Eicosanoids are crucially involved in several pathophysiological conditions [139], and the intersection between changes in certain platelet-derived eicosanoids and cancer appears particularly intriguing. For example, a multi-omics analysis of serum from metastatic melanoma patients revealed a rise in the concentration of 12-hydroxyeicosatraenoic acid (HETE) and 15-HETE eicosanoids, which are respectively produced by platelet 12-LOX and COX-1 [99]. These two platelet-derived eicosanoids have also been shown to exert pro-malignant effects in several cancer types, by activating mitogen and angiogenic pathways [139,140,141,142]. Coherently, blockage of COX-1 by aspirin causes the loss of platelet ability to transform human colon carcinoma cells into mesenchymal-like cells [139,143] and the long-term use of low-dose aspirin is associated with a reduction in risk of various cancers [144].

Finally, activated platelets release lysophosphatidic acid (LPA), a bioactive lipid growth factor, which has been shown to promote cell proliferation, survival, migration, tumor cell invasion and reversal of differentiation, through multiple G protein-coupled receptor (LPA1-6) cascades [145]. Several studies have found a relationship between plasma LPA levels and ovarian carcinoma; for example, a meta-analysis, comparing LPA levels in the serum of 980 ovarian cancer patients, 872 benign controls and 668 healthy controls, showed higher LPA plasma levels in the cancer group with respect to the benign and healthy control samples [146], thus suggesting that the raised detection of plasma LPA might be a potential diagnostic biomarker. Nonetheless, this finding is not supported by a recent lipidomic study that did not find any change in the content of this lipid in serum of ovarian cancer patients [147]. Discrepancies in the results can be explained taking into the account that, due to its susceptibility to sample processing procedure (e.g., plasma storage time at room temperature and anticoagulant used for blood drawing), LPA can artificially increase. In the light of this finding, therefore, it appears important to consider these confounding factors, in order to reduce to the minimum potential errors in measuring plasma LPA.

## 4. Platelet Microvesicle-Derived miRNAs

Upon activation, platelets release microvesicles (MVs), which are vesicular fragments with a diameter ranging from 0.5 to 1 μm, that express parental antigens (such as P-selectin and integrin αIIbβ3) and contain a plethora of mediators (growth factors, cytokines, inflammatory molecules, mRNAs and miRNAs) able to exert biological effects. These platelet MVs, accounting for 70–90% of all MVs circulating in the bloodstream, contribute to regulation of the tumor microenvironment and cancer-cell interactions [148,149,150]. Accordingly, cancer patients usually show increased levels of circulating MVs (in a way, that is different depending on tumor type, but it is directly proportional to tumor stage), which may be prognostic for monitoring tumor progression and response to specific therapeutics [151].

Although the role of MVs in cancer progression is multi-faceted and not fully understood, nonetheless it is becoming clear that MVs represent one of crucial determinants in tumor biology. Firstly, surface expression of platelet antigens leads to shedding of MVs displaying pro-coagulant and pro-thrombotic features. Therefore, MVs, together with activated platelets, enhance coagulation (which is further exacerbated by cancer-triggered activation of more platelets), thus playing an additional role in cancer progression [152]. Secondly, MVs are able to enhance angiogenesis: They stimulate the expression of pro-angiogenic molecules [including matrix metalloproteinase (MMP)-9, VEGF, IL-8 and hepatocyte growth factor (HGF)] in tumor cells [153] and drive capillary tube formation by stimulating endothelial cells [27,82,154].

Noteworthy, a bidirectional effect occurs as cancer cells can induce platelet activation and MV release; subsequently, a paracrine positive feedback mechanism is established, since MVs, taken up by cancer cells, potentiate the invasive phenotype through stimulation of migration [148,155]. Interestingly, although different cancer cells are able to induce platelet-derived MV release, only the most aggressive ones are responsive to MV action and, furthermore, only some subsets of MVs can positively feed back to cancer cells [155]. These findings suggest that i) cancer/platelet interplay is complex and strongly dependent on features of tumor cell type and ii) composition of MVs may differ depending on the stimulus given to platelets [155]. These findings may clarify discrepancies observed by authors in studies using different platelet preparations, agonists and cancer cell types. Just as an example, our recent work [156] showed that MVs, once internalized by cancer cells, inhibit migration rather than enhancing invasive properties; this may be explained considering that MV release was induced not by cancer cells but, instead, by a different stimulus (namely, arachidonic acid) that led to MV enrichment of specific bioactive molecules. Therefore, depending on MV composition, the effects on tumors may be completely different. A further point of discrimination may reside in MV internalization, since we found that, albeit taken up, the bioactive molecules delivered to cancer cells had different stability and, thus, exert their action in a time-and concentration-dependent fashion [156].

Among the bioactive molecules contained inside MVs, microRNAs (miRNAs) deserve a particular mention. Human platelets, indeed, contain an abundant repertoire of miRNAs that are released through MVs; depending on the nature of the agonists or stimuli activating platelets, the miRNA content of MVs can vary, but it always mirrors the content found in the platelets from which MVs derive [157]. It is exactly this heterogeneity of composition that may account for the observed differences in terms of molecular targets, mechanisms of action and effects on cancer cells (Table 3) [152]. If, on one side, cancer-promoting effects of MVs have been described (especially related to their content in growth factors, inflammatory cytokines and angiogenic factors), it is also true, on the other hand, that the ability to deliver miRNAs to recipient cells (including cancer cells) suggests a potential tumor-suppressive role. 

Platelet MVs can, indeed, be viewed as intercellular carriers that transfer inside cells specific molecules able to negatively modulate gene expression, with both positive and negative consequences. In this context, it should be underlined that most of the studies reporting differential effects of specific miRNAs in cancer employed transfection experiments, where miRNA expression was artificially increased and, to the best of our knowledge, only few of them checked the effects in more physiological (delivery of platelet-derived MVs) conditions.

Transfer of miRNAs to target cells has been shown to promote tumor progression [157]: miR-939 delivered by platelet MVs induces, in ovarian cancer cells, epithelial to mesenchymal transition, by down-regulating E-cadherin and up-regulating vimentin expression [163], while miR-223 has been shown to stimulate lung cancer cell invasion, by targeting tumor suppressor EPB41L3 [158]. Besides targeting tumor suppressor genes and oncogenes, several miRNAs enriched in MVs (miR-223, miR-24, miR-27a, miR-155, miR-195, let-7a/b) may also be implicated in therapy resistance. In small-cell lung cancer, miR-24-3p contributes to resistance to combination therapy (etoposide plus cisplatin), by targeting the autophagy-associated gene 4A [164]; other miRNAs that may be involved in drug resistance include miR-130a (which targets the pro-metastatic macrophage colony-stimulating factor (M-CSF)), and miR-27a and miR-451 (which target the multi drug resistance transporter 1) [166,167,172,173].

MVs also deliver angiogenic signals [27]: Transfer of miRNA let-7a or miR-27b in endothelial cells down-regulates the expression of the anti-angiogenic modulator thrombospondin-1, thus enhancing platelet-dependent endothelial tube formation [168,169].

However, what the available literature data suggest is that platelet-derived MVs may support cancer progression and metastatic dissemination at late stages, while it seems likely that they exert tumor suppressive roles at earlier stages. Michael’s group found that circulating MVs directly infiltrating lung and colon cancer cells deliver miR-24 that suppress tumor growth; this miRNA localizes to mitochondria where it inhibits mt-Nd2 and Snora75, resulting in mitochondrial dysfunction and induction of apoptotic cell death [165]. Similarly, miR-223 inhibits migration, stimulates anoikis cell death and enhances chemo-sensitivity in different cancer cell types [159,160,161]. We and others have demonstrated that also platelet-specific miR-126 exert tumor suppressive roles; this miRNA may be a predictor for tumor relapse in postmenopausal breast cancer patients treated with tamoxifen [174], impairs cancer progression through direct repression of MMP-9 [170], and MV-mediated delivery into breast cancer cells induces cell cycle arrest, inhibition of migration and sensitivity to cisplatin [156]. Finally, miR-126 and miR-223 exert antagonistic effects on angiogenesis: miR-126 stimulates VEGF-induced proliferation in endothelial cells [171], while miR-223 exerts an inhibitory effect on formation of new blood vessels, by targeting endothelial β1 integrin [162].

The ability of MVs to acquire distinct roles, depending on their repertoire of proteins and miRNAs, suggest that they may be used as biomarkers with diagnostic and therapeutic implications [175]. For example, plasma levels of platelet MVs, together with VEGF, IL-6 and RANTES, have been found to be increased in patients with stage IV gastric cancer [176]. Elevated amounts of endothelial and platelet MVs (that significantly decreased after chemotherapy) have been found in the plasma of non-small cell lung cancer patients, thus suggesting a predictive role for prognostic clinical outcome [177].

## 5. Conclusions

Although some symptoms of cancer, such as breast lumps, are classic “alarm” symptoms, others are ambiguous and more likely caused by other conditions. Accordingly, different studies demonstrated that alterations in platelet number or/and activity often occur in cancer patients. This finding, together with the evidence that platelets may basically affect all steps of tumor development, prompts researchers to carry out more studies for fully understanding the mechanisms underlying cancer-related platelet dysfunction.

**Figure 1:**
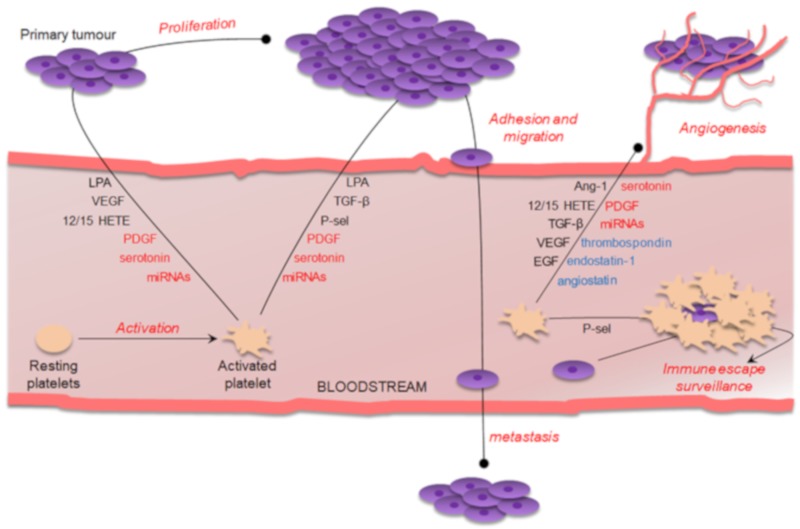
Schematic representation of the main platelet effects on tumor biology. See text for details. In black: platelet-derived bioactive molecules with positive effects. In blue: platelet-derived compounds with negative effects. In red: platelet-derived compounds with both positive and negative effects. Lines with dot indicate either stimulation or inhibition, depending on the platelet-derived bioactive molecule. 12/15 HETEs: 12 and 15 hydroxyeicosatraenoic acid; Ang-1: Angiopoietin; LPA: lysophosphatidic acid; EGF: Endothelial Growth Factor; P-sel: P-selectin; PDGF: Platelet-Derived Growth Factor; TGF-β: tumor growth factor-β; VEGF: vascular endothelial growth factor.

Environmental cancer-related stimuli encountered by platelets are intricate, as are the intracellular signaling pathways regulating platelet responses to the stimuli themselves. Moreover, due to heterogeneity in the cargo of growth factors, cytokines, microRNAs and other bioactive molecules and platelets may potentially release either stimulators or inhibitors in all cancer steps (Figure 1).

Accordingly, on one hand, there are a great deal of proofs of deadly interaction between platelets and cancer cells, but, on the other hand, some experimental and clinical data also indicate a protective role. Besides to complexity of platelet signaling in cancer, the scenario is further complicated by other confounding factors extrinsically related to platelets: (i) most of results come from retrospective studies, analyzing a wide range of patients with heterogeneous characteristics, such as age, sex, race, cancer type and stage, as well as treatments not always related to cancer; (ii) some studies have been carried out in cancer patients post-diagnosis and changes in platelets have not been monitored with respect to cancer progression over time, so that it is difficult to establish the exact contribution of platelets and if their changes are the cause or rather a consequence of tumor; and (iii) some of the bioactive compounds, whose plasma concentrations have been shown to be correlated with cancer progression, are also released by other cells, including cancer cells themselves; therefore, the effect on cancer, whether positive or negative, is not necessarily due to platelets.

Other questions still need to be answered: Why do platelet changes occur in some types of cancer and not in others? How does the platelet–cancer relationship change with age, sex and cancer progression over time? Although the heterogeneity and adaptive potential of the tumor features make a “one-size-fits-all” approach for targeting platelet–cancer interactions difficult, a better understanding of the interplay might provide efficient tools for cancer prevention, screening, risk assessment and management.

## Figures and Tables

**Table 2:** Main platelet-derived proteins involved in cancer.

Molecule	Main Findings	Role in Cancer	Ref.
**P-selectin**	↑ tumor cell extravasation by promoting cancer cell interaction with platelets and endothelium ↑ platelet activation by increasing thrombin generation ↑ monocyte TF exposure	**NEGATIVE**	[98,100,101,102,103]
**TF**	Clotting cascade activation ↑ cancer cell survival, ↑ angiogenesis, ↑ tumor growth, ↑ metastasis		[100]
**VEGF**	↑cancer growth and angiogenesis ↑ MK maturation		[101]
**EGF**	↑ mesenchymal and epithelial cell proliferation ↑ pro-angiogenic effect of other cytokines		[102]
**Ang-1**	↑ vessel development and maturation		[103]
**PDGF-BB**	↑ cancer cell proliferation, survival and invasion ↑ tumor stroma changes ↑ blood vessel maturation		[104,105]
	↓ tumor cell growth and dissemination ↓ metastasis	**POSITIVE**	[106]
**Endostatin,** **TSP-1,** **angiostatin**	↓ angiogenesis		[107,108]

Ang-1: angiopoietin-1; EGF: endothelial growth factor; MK: megakaryocytes; NK: natural kill cells; PAI-1: plasminogen activator inhibitor-1; PDGF-BB: Platelet-derived growth factor BB; TF: tumor necrosis factor; TSP-1: thrombospondin-1; VEGF Vascular endothelial growth factor.

**Table 3:** Main findings on the cancer-related role of miRNAs, potentially delivered by platelet MVs.

miRNA	Experimental Settings	Main Findings	Targets	Ref.
**miR-223**	Platelet MV delivey to lung A549 cancer cells	↑ cell invasion	EPB41L3	[158]
	Transfection of breast MCF-7 and prostate PC-3 cancer cells	↓ vitality, ↑ effects of the anti-tumor celastrol	NF-κB	[159]
	Transfection of breast MDA-MB-231 and MCF-7 cancer cells Incubation of MDA-MB-231 cells with CM derived from stable transduced MEFs or HEK293 cells	↓ migration, ↑ anoikis cell death, ↑ sensitivity to chemotherapy	STAT5A	[160]
	Transfection of breast MCF-7, SKBR3, MDA-MB-231 and MDA-MB-435 cancer cells	↑ sensitivity to TRAIL-induced apoptosis	HAX-1	[161]
	Transient transfection of primary endothelial cells	↓ formation of new blood vessels	endothelial β1 integrin	[162]
**miR-939**	Platelet MV delivey to ovarian SKOV3 cancer cells	↑ epithelial to mesenchymal transition	E-cadherin and vimentin	[163]
**miR-24-3p**	Transfection of small-cell lung H446 cancer cells	resistance to etoposide plus cisplatin therapy	ATG4A	[164]
**miR-24**	Platelet MV delivey to Lewis lung and colon MC-38 carcinoma cells	↓ tumor growth, ↑ apoptosis	mt-Nd2 and Snora75	[165]
**miR-130a**	miRNA microarray in drug-resistant ovarian A2780 carcinoma cells Transfection of cervix HeLa carcinoma cells	drug resistance	M-CSF	[166]
**miR-27a, miR-451**	Transfection of MDR ovarian A2780 and cervical KB-V1 carcinoma cells		MDR1	[167]
**miR-let-7a,** **miR-27b**	Platelet MV delivey to primary endothelial cells	↑ endothelial tube formation	thrombospondin-1	[168,169]
**miR-126**	Transfection of breast MDAMB231 and MCF7 cancer cells	↓ cancer progression	ADAM9	[170]
	Transfection of breast BT-549 cancer cells Platelet MV delivey to breast BT-549, MDA-MB-468, BT-20 and MCF-7 cancer cells	cell cycle arrest, ↓ migration, ↑ sensitivity to cisplatin	ND	[156]
	Transfection of lung A549, Y-90 and SPC-A1 carcinoma cells	↑ proliferation	VEGF	[171]

ND: not determined. ADAM9: ADAM Metallopeptidase Domain 9; ATG4A: autophagy-associated gene 4A; CM: conditioned medium; EPB41L3: Erythrocyte Membrane Protein Band 4.1 Like 3; HAX-1: HS-1-associated protein X-1; β; M-CSF: macrophage colony-stimulating factor; MDR1: multidrug resistance gene; MEF: mouse embryonic fibroblasts; mt-Nd2: Mitochondrial NADH dehydrogenase 2; MV: microvesicle; NF-κB: nuclear factor-κB; Snora75: Small Nucleolar RNA, H/ACA Box 75; STAT5A: signal transducer and activator of transcription 5A; TRAIL: TNF-related apoptosis-inducing ligand; VEGF: vascular endothelial growth factor.

## Abbreviations

Ang-1	Angiopoetin-1
COX	Cyclooxygenase
EGF	Epidermal growth factor
G-CSF	Granulocyte colony-stimulating factor
GM-CSF	Granulocyte-macrophage colony-stimulating factor
GRO	Growth-regulated oncogene
HETE	Hydroxyeicosatraenoic acid
HGF	Hepatocyte growth factor
HOA	Homeobox
HR	Hazard ratio
JAK	Janus kinase
IBC	Inflammatory breast cancer
IL	Interleukin
LOX	Lipoxygenase
LPA	Lysophosphatidic acid
M-CSF	Macrophage colony-stimulating factor
miRNA	MicroRNA
MMP	Matrix metalloproteinase
MV	Microvesicle
PDGF	Platelet-derived growth factor
PUFA	Polyunsaturated fatty acid
SNP	Single nucleotide polymorphisms
STAT	Signal transducer and activator of transcription
TF	Tissue factor
TGF	Transforming growth factor
TPO	Thrombopoietin
VEGF	Vascular endothelial growth factor
VTE	Venous thromboembolism
